# Advanced multifunctional hydrogels for diabetic foot ulcer healing: Active substances and biological functions

**DOI:** 10.1111/1753-0407.13537

**Published:** 2024-04-10

**Authors:** Yuetong Li, Yuxin Leng, Yang Liu, Jianhua Zhong, Jiaxin Li, Shitong Zhang, Zhenlin Li, Kaming Yang, Xinyi Kong, Wanwen Lao, Changlong Bi, Aixia Zhai

**Affiliations:** ^1^ Department of Endocrinology, The Eighth Affiliated Hospital Sun Yat‐sen University Shenzhen China; ^2^ Department of Critical Care Medicine Peking University Third Hospital Beijing China; ^3^ Department of Laboratory Medicine, The Eighth Affiliated Hospital Sun Yat‐sen University Shenzhen China; ^4^ Department of General Practice, The Eighth Affiliated Hospital Sun Yat‐sen University Shenzhen China

**Keywords:** biological functions, classification, diabetic foot ulcer, hydrogels, pathogenesis

## Abstract

**Aim:**

Hydrogels with excellent biocompatibility and biodegradability can be used as the desirable dressings for the therapy of diabetic foot ulcer (DFU). This review aimed to summarize the biological functions of hydrogels, combining with the pathogenesis of DFU.

**Methods:**

The studies in the last 10 years were searched and summarized from the online database PubMed using a combination of keywords such as hydrogel and diabetes. The biological functions of hydrogels and their healing mechanism on DFU were elaborated.

**Results:**

In this review, hydrogels were classified by their active substances such as drugs, cytokines, photosensitizers, and biomimetic peptide. Based on this, the biological functions of hydrogels were summarized by associating the pathogenesis of DFU, including oxidative stress, chronic inflammation, cell phenotype change, vasculopathy, and infection. This review also pointed out some of the shortcomings of hydrogels in present researches.

**Conclusions:**

Hydrogels were classified into carrier hydrogels and self‐functioning hydrogels in this review. Besides, the functions and components of existing hydrogels were clarified to provide assistance for future researches and clinical applications.

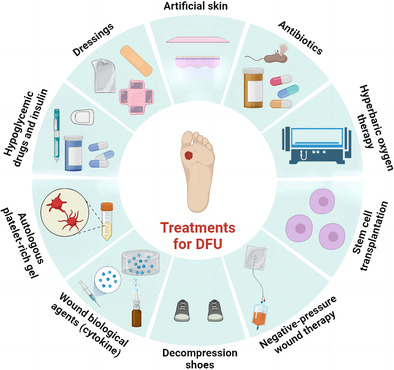

## Graphical abstract

Highlights

1. A review was conducted of studies on the combination of hydrogels and diabetic foot ulcer pathogenesis.
2. Various hydrogels were classified and elaborated based on their biological functions.
3. The summary of biological functions provides guidance for future research and clinical application.

## 1 INTRODUCTION

Diabetic foot ulcer (DFU) is the main source of pain and economic burden for diabetic patients and also one of the reasons for their disability and death.^1^, ^2^ Studies indicated that the global prevalence rate of DFU is 6.3%, and it is estimated that 9.1 million to 26.1 million diabetic patients worldwide suffer from foot ulcers.^3^, ^4^ DFU leads to amputation and has a high rate of amputation mortality with the 5‐year survival rate of only 41%–48%. Even for DFU patients with minor amputation, the 5‐year survival rate is only 59%.^5^ In addition, the recurrence of DFU is common. The recurrence rate after DFU healing is about 40% within 1 year, 60% within 3 years, and 65% within 5 years.^4^ The long‐term impact and recurrence of disease has brought huge social medical and economic burdens to world health.^6^

**FIGURE 1:**
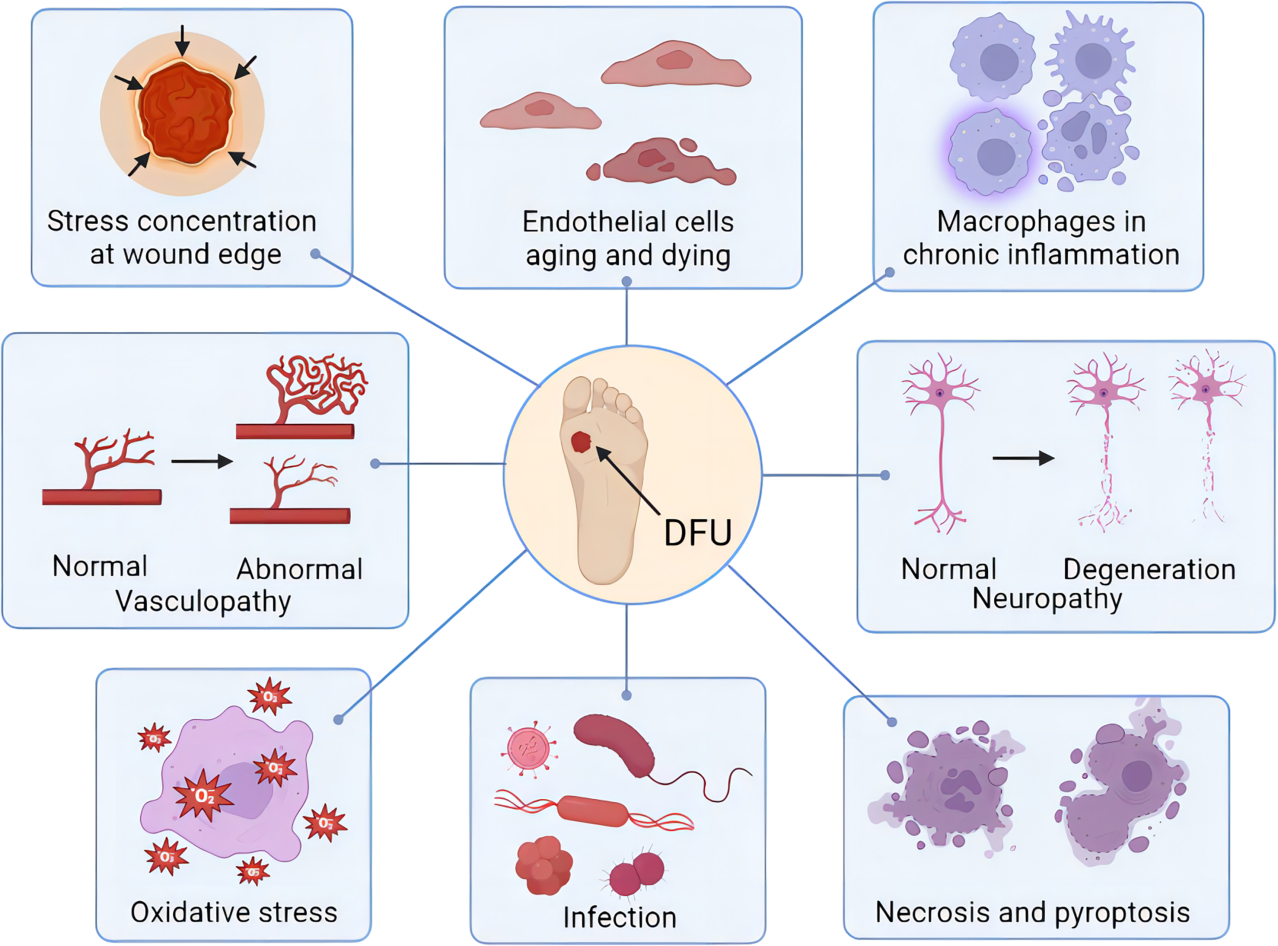
The pathogenesis of diabetic foot ulcer (DFU).

The pathogeneses of DFU including wound stress concentration, cell death, chronic inflammation, vasculopathy, neuropathy, oxidative stress, and infection are shown in Figure 1. Multiple factors lead to the onset and the difficulty in the healing of DFUs, including peripheral neuropathy, excessive oxidative stress, chronic inflammation, cell phenotype change, vasculopathy, infection, and other factors. These factors will cause slow regeneration of vessels and nerves around the wound, slow cell proliferation, and stagnation of inflammatory phase.^7^ For the pathogenesis of DFU, insulin resistance and hyperglycemia cause activation and antioxidants reduction of pathophysiological pathways, leading to oxidative stress and inflammation. Persistent oxidative stress and chronic inflammation lead to cellular damage, endothelial dysfunction, neuropathy, and angiopathy.^8^ Ultimately, all these factors lead to delayed wound healing, amputation, or death.

**FIGURE 2:**
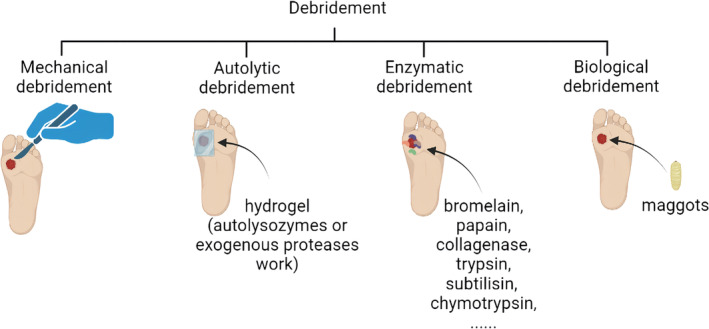
Wound debridement for diabetic foot ulcer (DFU).

**FIGURE 3:**
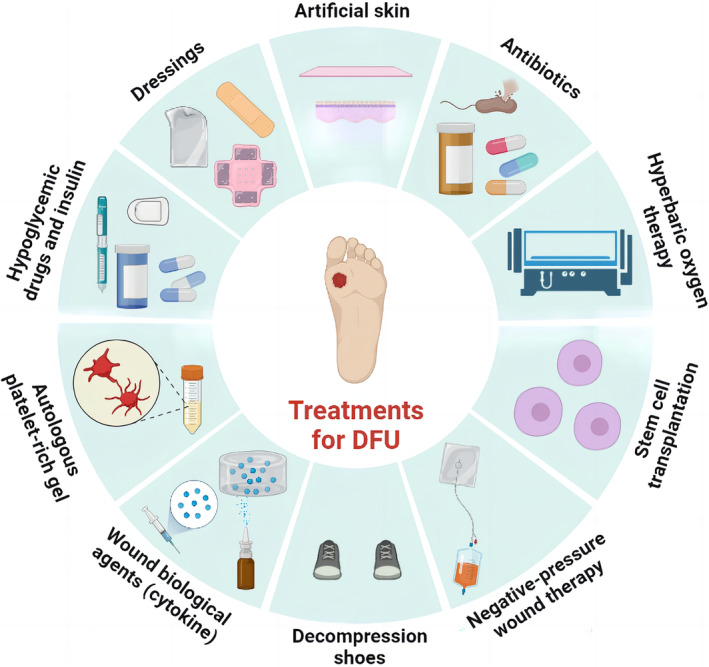
Current treatments for diabetic foot ulcer (DFU).

Over the past decades, important principles have been established in DFU treatment: debridement, burden reduction, antibiotics against infection, and closure with dressings.^9^ The types of wound debridement include mechanical debridement, autolytic debridement, enzymatic debridement, and biological debridement, which are described in detail in Figure 2. In addition to debridements, treatments for DFU, including lowering blood sugar, covering dressings, transplanting of artificial skin, using antibiotics, hyperbaric oxygen therapy, stem cell transplantation, negative‐pressure therapy, decompression shoes equipment, and cytokine and autologous platelet‐rich gel use, were summarized in Figure 3. Furthermore, wound closure is an important part of the therapeutic process of DFU. It is essential for a good dressing to close the wound, prevent bacterial invasion, simulate the extracellular matrix (ECM) environment, promote wound healing, and reduce the risk of amputation or death eventually.^10^, ^11^ However, the therapeutic effect remains unsatisfactory and most therapies still end with amputation or death presently. This is because traditional treatments do not target the pathogenesis of DFU specifically. Besides, the most widely used dressings in clinical practice, including skimmed cotton gauze and petroleum jelly gauze, cannot be loaded with drugs or mimic ECM, as well as prevent microbial invasion.^12^ Therefore, it is necessary to seek for more effective and suitable dressings based on the pathogenesis of DFU.

To solve these problems of the traditional dressings, advanced dressings such as hydrocolloids, hydrogels, foams, and films have been developed. Wet dressings can promote cell migration, proliferation, and collagen synthesis, which promote wound healing.^13^, ^14^ Among them, hydrogels stand out due to their unique advantages. Hydrogels can mimic the natural ECM environment, leading to the excellent biocompatibility and biodegradability. Furthermore, hydrogels can be used as drug carriers due to their porous structure and can lead to better therapeutic effects by loading with drugs or bioactive factors. More important, hydrogels have the ability to repond to stimulation. External stimulation (light and mechanical force) as well as internal stimulation (glucose and pH value) can induce hydrogels to release substances to play a role.^13^, ^15^, ^16^

It is reported that hydrogels close the wound and prevent microbial invasion due to their mechanical properties, such as stretchability and adhesion.^10^ However, the poor mechanical properties of the natural hydrogels limit their application. As for the synthetic hydrogels, although they have better mechanical properties due to their chemical cross‐linked property, their poor biodegradability and potential toxic effect remain major concerns.^17^ In addition, the encapsulation of hydrophobic substances of the hydrogels has poor efficiency, leading to their uncontrolled explosive release. Besides, the deformation and degradation of hydrogels lead to drug leakage, which compromises the therapeutic efficacy and even contribute to toxicity.^18^ These disadvantages result in the weak controllability and responsiveness of hydrogels.

For further clinical application, it is necessary to review the existing biological functions of hydrogels. It is also crucial to point out their shortcomings for the future improvement of hydrogels.

## 2 PATHOGENESIS OF DFU

The pathogenesis of DFU is particularly important for the study of the biological functions of corresponding hydrogels, and thus the pathogenesis of DFU is summarized as follows.

### 2.1 Peripheral neuropathy

Peripheral sensory neuropathy, the primary cause of DFU, can make patients unaware of trauma.^19^, ^20^ Among them, motor neuropathy can cause the anterior tibial muscle atrophy and foot deformity, resulting in abnormally high plantar pressure, following by the occurrence and development of foot ulcers.^21^, ^22^, ^23^ Besides, autonomic neuropathy often leads to anhidrosis of lower limbs and makes the foot skin dry and chapped, thus providing a channel for bacterial invasion.^24^, ^25^ The healing rate of ulcers in patients with peripheral neuropathy of the lower limbs will be significantly reduced because the neuropathy reduces the production of neuropeptides that promote cell proliferation and wound healing, resulting in the low cure rate and even amputation in patients with DFU.^8^ Additionally, the treatment of diabetic foot infection could also be ineffective because of poor neurological functional status.^26^, ^27^ Nevertheless, peripheral nerves can be given a supportive environment to regain their structure and function using the hydrogels loaded with growth factors and cytokines.^28^ Furthermore, by triggering signaling pathways, hydrogels containing metal ions can also encourage the regeneration of peripheral nerves.^29^

### 2.2 Oxidative stress

Under normal circumstances, physiological levels of reactive oxygen species (ROS) are essential for the process of wound healing. ROS contribute to bacterial death, platelet aggregation, revascularization, and reepithelialization. However, excessive ROS can induce oxidative stress and thus hinder wound healing by causing protein modification, lipid peroxidation, DNA damage, and finally cell apoptosis.^30^ Due to the hyperglycemic environment, DFU wounds show a high level of ROS and superoxide produced mainly by mitochondrial electron transport chain, which further promote oxidative stress injury and induce the production of advanced glycosylation end products (AGEs).^31^, ^32^ Meanwhile, the uncoupling of nitric oxide synthase leads to the reduced production of nitric oxide, which also leads to chronic inflammation in diabetic wounds.^33^, ^34^ In conclusion, high ROS delay the inflammatory process, hinder cell proliferation, and obstruct remodeling during the four stages of wound healing, including hemostasis, inflammation, proliferation, and maturation.^35^ Chronic inflammation and AGEs associated with ROS in DFU healing are elaborated in the following sections.

### 2.3 Chronic inflammation

**FIGURE 4:**
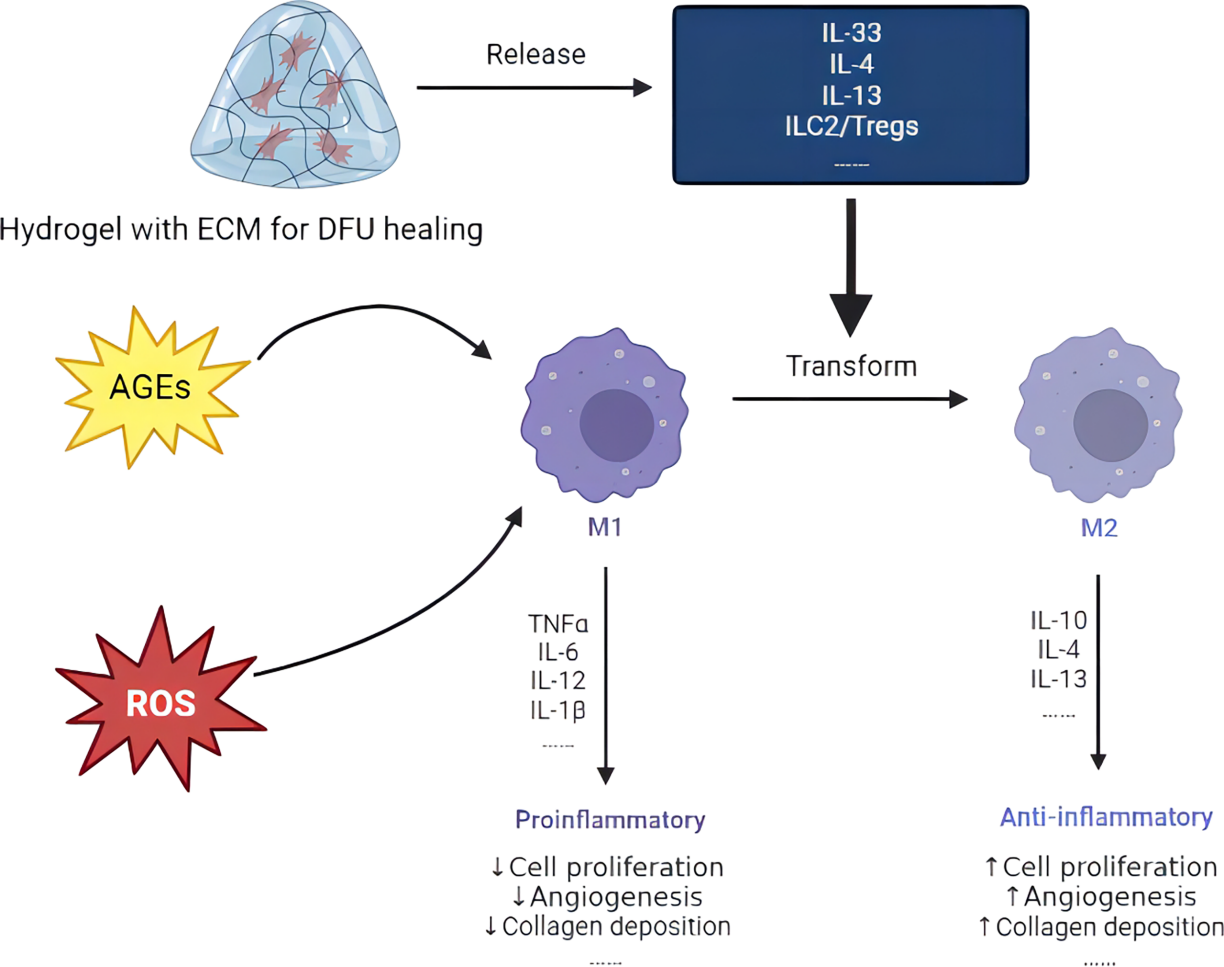
The immune regulation of macrophages. AGEs, advanced glycosylation end products; DFU, diabetic foot ulcer; ECM, extracellular matrix; IL, interleukin; ROS, reactive oxygen species; TNFα, tumor necrosis factor alpha.

During the stage of normal wound inflammation, macrophages change from an inflammatory phenotype to a repair supporting phenotype, as well as release various cytokines and growth factors.^35^ However, macrophages are still in an inflammation stage and cannot be transformed into a repair phenotype, leading to the inability to secrete the medium to promote tissue repair in the damaged tissues of diabetic patients. Moreover, the wound cannot turn to the proliferation stage, leading to chronic inflammation and nonhealing diabetic wounds.^34^, ^36^, ^37^ To be more precise, a decrease in M2 macrophages and an increase in the M1/M2 ratio lead to a deficiency in growth factors, such as epidermal growth factor (EGF), fibroblast growth factor (FGF), platelet‐derived growth factor (PDGF), vascular endothelial growth factor (VEGF), anti‐inflammatory cytokines like interleukin 10 (IL‐10), transforming growth factor (TGF)‐α, and TGF‐β. These elements are essential for both the proliferation and remodeling phases.^36^ Therefore, the regulation of the phenotype of macrophages is a significant way to diminish inflammation and facilitate healing. The combined impact of the regulation of the phenotype of macrophages with antioxidant stress generates a better therapeutic effect on DFU. Figure 4 is a sketch map that shows the regulatory effect of hydrogel on the phenotype of macrophages. In this graph, a hydrogel can release cytokines to accelerate the transformation from M1 macrophages. M1 macrophages remain chronic inflammation induced by ROS and AGEs, whereas M2 macrophages can promote proliferation and remodeling.

### 2.4 Vasculopathy

Vasculopathy is another important factor in the process of DFU. The reduced angiogenic factors and the increased antiangiogenic factors lead to the reduction of neovascularization, which prevents wound healing.^38^ In addition, the reduction of proportion of angiopoietins‐1/angiopoietins‐2 leads to the reduction of bone marrow endothelial progenitor cells. This transformation results in the impairment of the neovascularization capacity in diabetic wound.^39^, ^40^ In general, the state of diabetes mellitus causes a large number of neovascularization defects such as insufficient angiogenesis. These defects occur in the early and late stages of wound healing, as well as affect the proliferation and maturation of blood vessels.^38^, ^41^

### 2.5 Infection

Infection is also an important factor leading to the amputation. Pathogens precipitate and proliferate in the wound, causing chronic inflammation and thus aggravating wound injury. At present, the primary treatment is the use of antibiotics. However, bacterial resistance makes antibiotic treatment ineffective and poses a threat to human health.^1^, ^42^, ^43^, ^44^ Thus, drug‐resistant bacteria are important factors leading to poor prognosis of DFU.

### 2.6 Others

The occurrence and development of DFU are caused by multiple factors. In normal wounds, damaged and contracted blood vessels result in wound hypoxia, keratinocyte migration, early angiogenesis, proliferation and clonal expansion of fibroblast, transcription and synthesis of key growth factors, as well as cytokines. Subsequently, fibroblasts aggregate at the injured site for the participation of the formation of early granulation tissue and the activation of the contraction process.^45^, ^46^ In DFU, fibroblasts may be aging due to the phenotypic changes, which leads to their weakened proliferative response to growth factors and then affects granulation tissue formation and wound healing.^47^, ^48^, ^49^ Due to hyperglycemia, the original healing process is impaired and the biological functions of many cells have changed.

The AGE accumulation, polyol pathway, protein kinase C pathway and hexosamine biosynthesis pathway are the four main pathways in the occurrence and development of DFU. These four pathways play an important role in oxidative stress and chronic inflammation of DFU. Besides, these pathways are therapeutic targets for many hydrogel‐loaded drugs. Targeting these pathways can delay the development of DFU and promote DFU healing.

## 3 CLASSIFICATION AND BIOLOGICAL FUNCTIONS OF HYDROGELS

In previous reviews, hydrogels were always classified by materials. This classification did not focus on the biological function of hydrogels. In this review, the hydrogels were classified by whether they are loaded with drugs or play a single role. The biological functions of hydrogels and their relationship to the pathogenesis of DFU were focused.

### 3.1 Hydrogel as a carrier

Hydrogels are ideal carriers and dressings because of their unique internal structure, high biocompatibility, and mechanical stability.^50^ Hydrogels achieve the localized release of medicinal compounds, while avoid rapid clearance by kidneys.^51^ Therefore, existing therapeutic molecules, such as antioxidants, bioactive substances, and metal ions, can play a better role in treating DFU. Among these therapeutic molecules, the hydrogels play a role in ROS scavenging, the main effect of antioxidants, which is an effective way to promote wound healing and subsequent processes. The ROS scavenging of the antioxidants can cause the cell proliferation and angiogenesis.^52^, ^53^ However, scavenging ROS cannot achieve the optimal effect along, especially for diabetic foot infection. Therefore, almost all the existing hydrogels loaded with substances have several functions. This part reviews the recent advances of hydrogels loaded with substances. The effective substances and biological mechanisms of hydrogels reviewed in this part are summarized in Table 1.

**TABLE 1:** Effective substances and biological mechanisms of hydrogels loaded with substances.

Polymers	Effective substances	Models	Results	References
Polyethylene glycol	Exosome secreted by ADSC into the MMP	STZ diabetic mice	Removed ROS from cells to promote wound healing	52
DNA‐IL‐33‐Cytogel	Antioxidant DNA strand	STZ diabetic mice	Scavenged ROS and promoted wound healing	60
Quaternized chitosan (QCS) and benzaldehyde‐terminated F108 (F108‐CHO)	CO	STZ diabetic mice	Consumed ROS and activated the expression of HO‐1, thus promoting the diabetic wound healing	61
Oxidized hyaluronic acid (OHA) and carboxymethyl chitosan (CMCS)	Au–Pt nanozyme	STZ diabetic rats	Provided a favorable microenvironment for the healing of DFU chronic wounds	78
Poly (d,L‐lactide)‐poly(ethylene glycol)‐poly(d,L‐lactide) (PDLLA‐PEG‐PDLLA) hydrogel	Prussian blue nanoparticles	STZ diabetic rats	Protected mitochondria from oxidative stress damage and improved DFU wound healing	56
Chitosan/heparin/poly (γ‐glutamic acid)	SOD	STZ diabetic rats	Eliminated ROS and promoted chronic wounds healing	54
Symbiotic Algae−Bacteria hydrogel	Hydrogen	STZ diabetic mice	Promoted cell proliferation and wound healing	62
Ulvan dialdehyde, chitosan, dopamine (DPA) and silver nanoparticles (Ag NPs)	The sulfate groups in ulvan and the catechol groups in dopamine	STZ diabetic mice	Scavenged DPPH, which is conducive to DFU wound healing	63
N‐carboxyethyl chitosan (N‐chitosan) and adipic acid dihydrazide (ADH) with hyaluronic acid‐aldehyde (HA‐ALD)	BM‐MSCs	STZ diabetic rats	Promoted granulation tissue formation, collagen deposition, cell proliferation, and angiogenesis	64
Inorganic Zn2 + −induced self‐assembled GA and photo‐crosslinked methyl acrylated silk fibroin (SF)	Glycyrrhizic acid	STZ diabetic rats	Reduced the activation of M1 macrophages, induced the transition to M2 phenotype, increased the expression of anti‐inflammatory and angiogenic factors, ameliorated the local wound microenvironment, and accelerated tissue remodeling	91
Porcine small intestinal submucosa‐based hydrogel	SC‐Ps‐sEVs	STZ diabetic rats	Promoted the proliferation and migration of mouse embryonic fibroblasts, collagen synthesis, and vascular reconstruction, thus promoting wound healing	67
			Increased the expression of HIF‐1α and VEGF, as well as activated HIF‐1α/VEGF and wnt4/β‐catenin pathway, to promote angiogenesis	
NaH2PO4, Na2HPO4, NaCl, and KCl	Peptide Jelleine‐1 and 8Br‐cAMP	STZ diabetic rats and mice	Accelerated the closure of cell‐free gaps and boosted cell proliferation, migration, and differentiation	86
Hyaluronic acid (HA) and chitosan	EGF and curcumin	STZ diabetic mice	Promoted granulation tissue formation and reepithelization	58
Graphene oxide (GO)‐based hydrogel	sEVs with miR‐21‐5p	STZ diabetic mice	Regulated PVT1/PTEN/IL‐17 axis, contributing to cell proliferation, migration and angiogenesis	71
Carboxymethyl chitosan‐based hydrogel	bFGF	STZ diabetic mice	Promoted fibroblast proliferation, ECM fibrosis, collagen formation, epidermal growth, hair follicle growth, and angiogenesis	69
Polydopamine/acrylamide (PDA/AM) hydrogel	MnO_2_ nanoparticles	STZ diabetic mice	Photothermal sterilization	79
Quaternized chitosan	GQDs‐ε‐PL, QCS, and high temperature	STZ diabetic rats	Destroyed bacterial membranes, leading to cytoplasmic outflow and bacterial death, and provided a closed environment for the wound to prevent reinfection and promote wound healing	81
PLGA‐PEG‐PLGA	Niobium carbide	Diabetic mice	Protected cells from oxidative stress damage, increased cell survival rate and boosted wound healing	83
Gelatin methacryloyl hydrogel	VH298	STZ diabetic mice	The enhanced blood supply and angiogenesis promotes wound healing effectively	70
Gelatin methacrylate (GelMA), adenine acrylate (AA), and CuCl2	Adenine acrylate and CuCl_2_	STZ diabetic mice	Regulated the levels of α‐SMA and CD31 to promote angiogenesis Played a critical antibacterial role in inhibiting inflammation and promoting wound healing	73
Keratin‐pullulan based hydrogel	CTX	STZ diabetic rats	Played an antibacterial role and promoted wound healing	85

Abbreviations: α‐SMA, alpha‐smooth muscle actin; ADSC, adipose‐derived stem cell; bFGF, basic fibroblast growth factor; BM‐MSC, bone marrow mesenchymal stem cell; CTX, cefotaxime sodium; DFU, diabetic foot ulcer; ECM, extracellular matrix; EGF, epidermal growth factor; HIF‐1α, hypoxia inducible factor‐1α; HO‐1, heme oxygenase‐1; IL‐17, interleukin‐17; MMP, matrix metalloproteinase; PTEN, phosphatase and tensin homolog; PVT1, plasmacytoma variant translocation 1; ROS, reactive oxygen species; sEV, small extracellular vesicle; SOD, superoxide dismutase; STZ, streptozotocin; VEGF, vascular endothelial growth factor.

#### 3.1.1 Hydrogels loaded with antioxidants

Oxidative stress is one of the most important pathogeneses of DFU. Therefore, many hydrogels are loaded with antioxidants that contribute to the reduction of excessive ROS. After eliminating the elevated ROS, the delayed inflammatory process can be promoted and the polarization of anti‐inflammatory macrophages can be induced. The proliferation and remodeling are then accelerated. Antioxidants include antioxidative enzymes and nonenzymatic antioxidants. The next sections str devoted to summarizing the development of hydrogels loaded with antioxidants on a category‐by‐category basis.

##### Antioxidative enzymes

Antioxidative enzymes mainly include superoxide dismutase (SOD), thioredoxin peroxidase, glutathione peroxidase, and catalase (CAT). A study demonstrated that SOD, an antioxidant enzyme, can facilitate the transformation of superoxide radicals (O^2−^) into H_2_O_2_, thereby expediting the healing of chronic diabetic wounds.^54^ In addition, a nanozyme hydrogel with the combination of SOD, CAT, glucose oxidase, peroxidase (POD), and nitric oxide synthase exhibited the synergistic enhancement effect of accelerating diabetic wound healing.^55^ This synergistic enhancement effect is achieved by reducing inflammation, relieving hypoxia, lowering blood glucose, promoting angiogenesis, and eliminating pathogenic bacteria.^55^ Besides, Xu et al^56^ developed a hydrogel loaded with Prussian blue nanoparticles, which can simulate the activity of CAT, POD, and SOD to eliminate ROS effectively.^57^ Besides, they can also restore the activity of nuclear factor erythroid 2‐related factor 2/heme oxygenase‐1 signal pathway. This finding suggests that enzyme mimics are also an effective therapy for DFU by scavenging excessive ROS.

##### Nonenzymatic antioxidants

Nonenzymatic antioxidants can be classified into natural and synthetic antioxidants. Previous studies reported that hydrogels loaded with natural antioxidants, such as curcumin, could eliminate oxidative stress and thereby promote wound healing remarkably.^58^, ^59^ DNA also acts as an antioxidant. Wang et al^60^ developed an antioxidant DNA hydrogel that can scour hydroxyl radical (· OH), a typical ROS. Besides, synthetic antioxidants designed by researchers emerge in an endless stream. Chen et al^61^ developed an all‐in‐one hydrogel that constantly releases CO by stimulating CORM‐401 (an oxidant‐sensitive CO‐releasing molecule) to consume ROS. The stimulation of CORM‐401 could lead to anti‐inflammation and antibacteria. Surprisingly, live single‐celled organisms can serve as antioxidants as well. Chen et al^62^ developed a symbiotic algae‐bacteria dressing that can produce hydrogen to resist oxidation and eliminate ROS and thus reduce inflammation. This hydrogel is composed of living *Chlorella vulgaris* (*Chlorella*) and *Bacillus licheniformis* (*Bacillus*), which produce hydrogen continuously for 60 h, as well as selectively reduced highly toxic ·OH and ONOO—. The addition of *bacillus* consumes excess oxygen and increase the hydrogen production, whereas *Bacillus* probably exacerbate the hypoxia of the wound environment and thus lead to the impairment of the wound healing.

#### 3.1.2 Hydrogels loaded with stem cells and cytokines

Hydrogels loaded with stem cells mainly play a role in cell proliferation and angiogenesis.^63^ Bai et al^64^ developed a self‐healing hydrogel loaded with bone marrow mesenchymal stem cells (BM‐MSCs). The molecules secreted by BM‐MSCs, such as TGF‐β1, VEGF and basic FGF (bFGF), can effectively promote the proliferation of fibroblasts, keratinocytes, and vascular endothelial cells.^65^ Additionally, BM‐MSCs can suppress M1 macrophages and stimulate the activation of M2 macrophages to alleviate chronic inflammation in the DFU wound microenvironment.^66^ In another study, MSC‐derived small extracellular vesicles (sEVs) were bound to the porcine small intestinal submucosa‐based hydrogel material through the peptides (SC‐Ps‐sEVs) to increase the content and achieve a continuous release.^67^ This study showed that sEVs can trigger the wingless/integrated (Wnt)/β‐catenin signaling pathways, and then up‐regulate the expression of their downstream proteins. It proved that SC‐Ps‐sEVs hydrogel promotes angiogenesis by activating hypoxia inducible factor‐1α/VEGF pathway. In addition, PDGF‐D is also proved to be an important factor in EV‐mediated angiogenesis.^68^ Moreover, the activation of wnt4/β‐catenin is another mechanism that promotes angiogenesis. However, the low survival rate and short acting time of stem cells are still the major problems for researchers. Therefore, improving the survival rate and action time of stem cells is an important direction to improve the performance of hydrogels loaded with stem cells in the future.

Cytokines also can act as an bioactive substances for cell proliferation and angiogenesis.^52^, ^54^, ^58^, ^60^, ^69^, ^70^ The carrier‐hydrogels play an important role in prolonging the release of cytokines and can protect cytokines from enzymatic degradation in the wound microenvironment due to its structure. Hao et al^69^ used carboxymethyl chitosan with better water solubility to combine with bFGFs and thus protect the bFGFs. This hydrogel loaded with bFGF can upregulate the expression of Ki67 to promote the growth of collagen fibers and epithelialization. Besides, it can also increase the expression of platelet endothelial cell adhesion molecule‐1 (PECAM‐1/CD31) and CD34 to promote angiogenesis and the formation of hair follicles.

In addition to directly loading stem cells and cytokines, there are many hydrogels loaded with inorganic or organic substances. This loading model can stimulate cells to secrete cytokines and thus promote cell proliferation and remodeling. Chen et al^71^ designed a hydrogel loaded with graphene oxide (GO). This hydrogel loaded with GO can induce sEVs secretion of miR‐21‐5p in adipose‐derived MSCs through up‐regulating the phosphoinositide‐3 kinase (PI3K)/protein kinase B (PKB/AKT) signal pathway. The MiR‐21‐5p can suppress the expression of phosphatase and tensin homolog deleted on chromosome 10 and plasmacytoma variant translocation 1 and thus promote proliferation, migration, and angiogenesis.

#### 3.1.3 Hydrogels loaded with bactericidal substances

Infection is an important cause of amputation and even death. Antibiotic resistance and side effects of systemic administration have been the serious problems of fighting infection. Based on this, advanced antibacterial hydrogels have been widely studied. Among them, hydrogels loaded with germicidal substances have shown excellent antibacterial activity in diabetic wound healing.^72^

##### Metal ions

It is extensively reported that metal ions have powerful bactericidal effect and have been used in clinical practice. To maintain the bactericidal effect, as well as avoid oxidation and aggregation, metal ions are combined with functional hydrogels.^72^ Although metal ions are biologically toxic, their healing effect is obvious and the drug resistance of them has not been developed. To counteract the biological toxicity of metal ions, other substances can be added into hydrogels. Chen et al^73^ developed an antibacterial adhesive self‐healing hydrogel loaded with CuCl_2_ and adenine acrylate. Cu^2+^ as well as other metal ions can destroy bacterial cell membranes and change the structures of proteins or enzymes to achieve a good antibacterial effect.^74^ Nevertheless, they found that Cu^2+^ may inhibit cell proliferation. Therefore, adenine, which can increase the stability of new blood vessels, was added to promote cell proliferation and angiogenesis.^75^

Except for Cu^2+^, the other metal ions such as Zn^2+^ can accelerate the proliferation and migration of fibroblasts and keratinocytes, thereby enhance tissue repair during wound healing.^76^, ^77^ In addition to bactericidal effect, many hydrogels loaded with metal ions can promote angiogenesis and scavenge ROS.^78^

##### Photosensitizers

Phototherapy is also a well‐known method of sterilization. Phototherapy includes photothermal therapy (PTT) and photodynamic therapy (PDT). PTT converts light into heat energy, and PDT leads to the generation of ROS to kill bacteria. Photosensitizers are added to hydrogels and irradiated with specific lasers to perform PTT and PDT. Traditional photosensitizers suffer from aggregation‐caused quenching effect, but advanced photosensitizers exhibit aggregation induced emission and stable property. Phototherapy also has its risks. PTT can heat up to 40–50°C, which may cause burns of the tissue.^79^, ^80^ PDT can increase the ROS load, which may aggravate oxidative stress damage in wound area. Therefore, phototherapy should be combined with other therapies to reduce the risk.^81^

Due to the piercing power of the laser, phototherapy has great potential in superficial tumors and skin wounds. Currently, antineoplastic and antibacterial effects of near‐infrared phototherapy are extensively studied. PTT is also applied in DFU. Common photothermal photosensitizers in DFU mainly include carbon‐based compounds, noble metal nanomaterials, and polymerics due to their excellent effect of photothermal conversion.^82^ Chen et al^83^ made a customized niobium carbide delivery hydrogel, which showed excellent antibacterial activity with near‐infrared photothermal conversion against *Staphylococcus aureus* (*S. aureus*) and *Escherichia coli* (*E. coli*). Under photothermal therapy (48°C), the thermosensitive niobium carbide nanosheets could kill most bacteria through the synergistic effect of internal antibacterial activity and photothermal bacterial ablation. Meanwhile, the thermosensitive niobium carbide nanosheets can effectively reduce the intracellular ROS induced by H_2_O_2_. However, as in most existing studies, the researchers did not evaluate the damage of the skin cells caused by low temperature. Therefore, this is a potential point for near‐infrared phototherapy to be paid attention to and studied in the future.

ROS generated by PDT can destroy the cell membrane and DNA of microorganisms to achieve microbicidal effect. The bactericidal effect of photosensitizers on Gram‐positive bacteria is greater than its effect on Gram‐negative bacteria due to the different membrane structure.^72^ The photosensitizers delivery systems are under intense research to improve hydrophilicity, reduce the side effect to normal tissues, and enhance the efficiency of PDT.^72^ Deep sterilization mechanism is lacking in both PTT and PDT. The therapeutic targets of heat or ROS have not been well studied. It is also not clear whether photosensitizers work outside or inside bacteria, and thus this problem deserves further study. However, there is no denying that phototherapy is a promising therapy in the future.

##### Antibiotics

Antibiotics are the major drugs for treating infection in clinical practice. However, drug resistance and systematic toxicity are developed due to abuse and inappropriate use.^84^ Therefore, in order to avoid abuse and systemic poisoning, hydrogels are loaded with antibiotics to achieve local delivery. Khaliq et al^85^ developed keratin‐pullulan based hydrogel membranes loaded with cefotaxime sodium (CTX). However, this study indicated that the hydrogel promotes wound healing mainly due to the keratinocyte migration promoted by keratin and pullulan whereas the antibacterial effect is only auxiliary. Nevertheless, this paper did not compare the antibacterial effect of the hydrogel loaded with CTX and direct application of antibiotics, nor did it study the possible drug resistance. Anyhow, it is believed that adding antibiotics to hydrogel is not a good antibacterial method.

##### Antibacterial peptide

Compared with antibiotics, antibacterial peptide without drug resistance may be a better choice to treat drug‐resistant bacteria. Natural antibacterial peptides Jelleine‐1 and 8Br‐cAMP were added to the hydrogel by Zhou et al.^86^ In addition to its antibacterial effect, Jelleine‐1 stimulates the secretion of growth factors such as bFGF, hepatocyte growth factor, TGF‐β, and VEGFA and thus promotes cell migration and angiogenesis.^87^ However, the high production costs and environmental sensitivity limit the application of natural antibacterial peptides. To reduce the cost and improve the stability, synthetic antibacterial peptides are being designed.^72^

### 3.2 Self‐functioning hydrogels

Some hydrogels circumvent drug leakage due to the complex procedures required to load substances. These hydrogels work on their own. The active components of hydrogels may be natural compounds, synthetic compounds, or biomimetic peptides. They have the effects of ECM environment simulation, sterilization, oxidative stress elimination, and anti‐inflammation. Besides, advanced self‐contractile hydrogels use biophysical properties to promote wound healing.^88^, ^89^ In the next section, the recent advances of self‐functioning hydrogels are reviewed and the effective substances and biological mechanisms of self‐functioning hydrogels are summarized in Table 2.

**TABLE 2:** Effective substances and biological mechanisms of hydrogels made from effective ingredients.

Polymers	Effective substances	Models	Results	References
Glucomannan (GM)‐P@Hyaluronic acid (HA)‐P hydrogel	Glucomannan	STZ diabetic rats	Triggered the intracellular signal transduction pathway related to M2 polarization, thus promoting wound healing by lessening inflammation and stimulating angiogenesis	97
Chitosan/sodium alginate/velvet antler blood peptides (CS/SA/VBPs) hydrogel	Velvet antler blood peptide	STZ diabetic mice	Promoted angiogenesis, cell proliferation, and ECM formation	95
A self‐assembling peptide (SAP) KGH hydrogel	KGH	STZ diabetic mice	Enhanced cell proliferation, granulation tissue formation, angiogenesis, and ECM remodeling	98
Polyacrylamide, gelatin, and ε‐polylysine	ε‐poly‐l‐lysine	STZ diabetic rats	Destroyed the membrane structure by interfering with the ζ potential of the surface, resisted the invasion of external bacteria and promoted wound healing	93
Ag‐ε‐polycaprolactone (PCL)/Gelatin‐methacryloyl (GelMA)	ECM‐mimic coaxial hydro‐membranes	STZ diabetic rats	Regulated surface immunomodulatory functions to accelerate DFU wound healing	88
Trans‐1,4‐cyclohexanediamine and 1,3‐dibromo‐2‐propanol	Cations	Male SD rats	Destroyed the bacterial membranes through electrostatic adsorption, thus killing the bacteria conducive to wound healing	89
Hydrophilic polyurethane and poly‐(acrylic acid) grafted with N‐hydroxysuccinimide ester (PAA‐NHS ester) and chitosan	The strain‐programmed patch	Diabetic db/db mice, human skin, pigs, and humanized mice	Contracted mechanically and programmatically, accelerating wound‐healing processes	99
l/d–phenylalanine derivatives	DH	STZ diabetic mice	Promoted cell migration and proliferation by affecting mechanical adjustment pathways	100
Gelatin methacryloyl and oxidized chondroitin sulfate (OCS)	OCS‐polypyrrole conductive nanoparticles	STZ diabetic rats	Promoted the migration of nerve cells and the regeneration of axonal by increasing intracellular Ca^2+^ concentration to activate PI3K/AKT and MEK/ERK signaling pathways, thus contributing to diabetic wound healing	105

Abbreviations: CTX, cefotaxime sodium; DFU, diabetic foot ulcer; DH, dextro‐chiral hydrogel; ECM, extracellular matrix; ROS, reactive oxygen species; SD, Sprague Dawley; STZ, streptozotocin.

#### 3.2.1 Self‐antioxidant hydrogels

Compared to hydrogels loaded with antioxidants, self‐antioxidant hydrogels avoid the incompleteness, instability, and explosive release of antioxidants. Besides, self‐antioxidant hydrogels avoid the regulatory obstacles of biological products and thus greatly shorten the review cycle and achieve clinical success.^59^ Some antioxidant macromolecules are quite suitable to form hydrogels. Zhu et al^90^ used an antioxidant macromolecule, poly(polyethylene glycol cocitric acid‐co‐N‐isopropylacrylamide) (PPCN), with thermoresponsive effect to form a hydrogel by regulating temperature. This study showed that peptide‐functionalized PPCN significantly reduced oxidative damage and promoted wound closure. It proved that the efficacy of self‐antioxidant hydrogels is not inferior to that of hydrogels loaded with antioxidants.

On the other hand, self‐antioxidant hydrogels can combine with other substances and perform other functions. It is reported that a glycyrrhizic acid (GA)‐based hybrid hydrogel can scavenge ROS and inhibit the inflammatory reaction.^91^ GA‐only hydrogel has poor mechanical property and inevitable cytotoxicity at high concentration, but Qian et al^91^ are the first to find that Zn^2+^ can solve these problems. With the synergistic effect of Zn^2+^, GA downregulates the nucleotide‐binding and oligomerization domain (NOD)‐like receptor signaling pathway to inhibit inflammatory reactions by lipopolysaccharide‐activated toll‐like receptor‐4 mediated signaling cascades. The signal transducer and activator of transcription (STAT) and interferon regulatory factor were included in the NOD‐like receptor signaling pathway. It is believed that self‐antioxidant hydrogels can provide more choices for the treatment of DFU.

#### 3.2.2 Self‐antibacterial hydrogels

The inherent antibacterial capability of self‐antibacterial hydrogels is because of their antibacterial components, such as antibacterial polymers, peptides, and amphoteric ions. Hydrogels with inherent antibacterial ability avoid the leakage of antibacterial substances and maintain the long‐lasting efficacy.^92^ As a natural antibacterial material, ε‐poly‐l‐lysine was used to develop a multifunctional hydrogel by Liu et al.^93^ This hydrogel also showed extensive temperature resistance, durability, and adhesiveness and can be used in extreme environment. Therefore, this hydrogel mainly kills bacteria by damaging the cell membranes and has a long‐lasting antibacterial performance in the temperature range of −20 to 60°C. Besides, self‐assembling peptides are recognized as an appropriate component of hydrogels. Bai et al^94^ developed an enzymatic A_9_K_2_ hydrogel with high antibacterial capability against both Gram‐positive and Gram‐negative bacteria. The amphiphilic peptide A_9_K_2_ associates with cell membrane and kills bacteria by membrane permeabilization. In addition to bactericidal effect, some hydrogels can improve the relative abundance of beneficial skin microbiota and reverse the structural imbalance of microbiota.^95^ To sum up, self‐antibacterial hydrogels are demonstrated to be a promising dressing to maintain the long‐lasting antibacterial efficacy without toxicity.

#### 3.2.3 Hydrogels as a 3D platform

A structural scaffold of cellular components plays an important role in cell proliferation, cell migration, tissue development, regeneration, and wound healing.^96^ Thus, researchers designed neotype hydrogels by construct a 3D microenvironment, which mimics the structure of ECM to promote DFU healing. This kind of hydrogel could regulate the expression of cytokines and activate certain signal pathways to promote wound healing.^97^ Liu et al^97^ developed an immunomodulatory glycopeptide hydrogel that can imitate the ECM. Glucomannan is a mannose receptor ligand with high affinity for macrophages. The glucomannan carried by the hydrogel induces the intracellular signal transduction pathway, which is related to M2 polarization of the macrophages. The macrophages convert into the M2 phenotype through the extracellular‐signal regulated kinase (ERK)/signal transducer and activator of transcription 6 (STAT6) pathway. A native 3D microenvironment like ECM in skin cells can significantly promote angiogenesis and tissue regeneration, in comparison to the conventional 2D culture.^98^ It demonstrated that hydrogels as a bioactive 3D platform can be a potential therapy for DFU.

#### 3.2.4 Hydrogels with physical properties

Increasing attention has been paid to the physical properties in wound healing. It is increasingly valued that unresolved stress concentration at the wound edge leads to the expansion of DFU wound. Therefore, mechanical modulation is a promising strategy to repair and remodel the skin.

To solve the problem of stress concentration at the wound edge, Theocharidis et al^99^ developed a strain‐programmed hydrogel patch, which can be classified into a bioadhesive layer and a nonadhesive elastic backing. The adhesive layer of the hydrogel patch can be adhered to the wound quickly and firmly without damaging the tissues. The elastic backing of the hydrogel patch can make the patch contract mechanically and programmatically through a hydration‐based shape memory mechanism. The patch is pre‐stretched proportionally while in the off‐body state, followed by dehydration to set the form and adhere to the wound. Finally, it rehydrates to contract and shrink a wound for healing.

In addition to stress concentration, it is known that the interactions of cells and ECM can have a huge impact on cell proliferation, migration, and differentiation. Zhu et al^100^ revealed the molecular mechanism of dextral chiral structures on promoting keratinocytes proliferation and developed a dextro‐chiral hydrogel (DH). They found that keratinocytes can sense the chiral changes in ECM via integrin *α*2*β*1 to alter their adhesion status on the external environment. Besides, DH can actively adsorb type I collagen, which provides DH with more cellular adhesion sites, and thus enhances local adhesion formation and cytoskeletal architecture.^101^, ^102^, ^103^, ^104^ Moreover, they provided evidence that chirality can activate YAP (actin cytoskeleton and integrin *α*2*β*1 activator) to promote keratinocyte proliferation via Hippo signaling pathway. Therefore, Zhu et al^100^ are the first to apply the chiral structures in dressings. However, the effect of cytoskeleton on the cell proliferation regulation of DH is not completely understood, and more studies are needed for further exploration on this point.

Conductivity is also an important physical property. It is reported that conductivity in skin tissue is essential for multiple biological processes, especially angiogenesis and nerve regeneration. Fan et al^105^ designed a conductive interpenetrating network hydrogel based on the ECM. This conductive hydrogel can promote protein phosphorylation in the mitogen‐activated protein kinase kinase (MAPKK/MEK)/ERK and PI3K/AKT pathways to promote angiogenesis and neural regeneration by increasing intracellular Ca^2+^ concentration. As is known, severe vasculopathy and peripheral neuropathy are the main causes of impaired DFU healing. Thus, promotions of angiogenesis and nerve regeneration are the key factors in DFU healing. Although more physical properties in wound healing are waiting to be discovered, more hydrogels with physical properties to promote DFU healing could appear.

## 4 CONCLUSION

DFU is a severe complication of diabetes mellitus and usually results in amputation or even death. Many researchers have developed various multifunctional hydrogels to promote wound healing based on the pathogenesis of DFU. Although most multifunctional hydrogels were not applied in clinical practice until now, the development in the next decade will provide clinicians with better treatment options and is worth expecting.^106^ The existing hydrogels can be mainly divided into those that act by loading substances and those that act on their own. They exhibit functions such as anti‐oxidation, anti‐inflammation, anti‐infection, and regulation of the cellular microenvironment, thereby promoting wound healing.

Of course, the research on multifunctional hydrogels has many issues and challenges. There is no consensus on the optimal animal model to represent human DFU. Besides, the lack of frontier research on large animals that are similar to humans and lack of the clinical trials limit the clinical applications of multifunctional hydrogels.^99^ On the other hand, many hydrogels are added with healing‐promoting active substances or drugs. However, the use of small molecules may depend on wound size, depth, and local vascularity. These factors are often difficult to estimate.^107^ In addition, the large amount of exudate from DFU wounds requires frequent hydrogel changes, which increases the cost for patients. It also suggests that hydrogels with the enduring ability to absorb exudate are needed.^108^ In conclusion, although studies on multifunctional hydrogels for DFU wounds still face pressing problems and challenges to be solved, it cannot be denied that they have tremendous clinical potential.

According to this review, multifunctional hydrogels have good performance. They have apparent effects on promoting wound healing in DFU, including antioxidative stress, immunomodulation, promotion of cell proliferation and migration, promotion of angiogenesis, and anti‐infection. These effects prove the possible clinical potential of multifunctional hydrogels. However, most studies did not discuss the deep mechanisms of their hydrogels to promote wound healing. Therefore, increasing discussions of their mechanisms of action should be included in the future. Moreover, with future research, the increase of the clinical trials and the advancement of clinical applications can reduce the amputation rate and mortality rate of DFU, as well as decrease the enormous medical and economic burden on patients and society.

## AUTHOR CONTRIBUTIONS

Yuetong Li and Yuxin Leng drafted the manuscript. Yang Liu, Jianhua Zhong, Jiaxin Li, and Shitong Zhang collected the related references and illustrated figures. Wanwen Lao, Xinyi Kong, Zhenlin Li, and Kaming Yang collected the related references and illustrated tables. Changlong Bi and Aixia Zhai conceived the project and edited the manuscript. All authors read and approved the final manuscript.

## FUNDING INFORMATION

This work was supported by the National Natural Science Foundation of China (82072267, 82272271, 82172215, and 81871562), Science and Technology Project of Guangdong Province (2021A1515011396, 2021A1515012109), and Shenzhen Futian District Public Health Research Project (FTWS2020017, FTWS2020008).

## CONFLICT OF INTEREST STATEMENT

The authors declare that the research was conducted in the absence of any commercial or financial relationships that could be construed as a potential conflict of interest.
